# Assessment of the Soil Organic Carbon Sink in a Project for the Conversion of Farmland to Forestland: A Case Study in Zichang County, Shaanxi, China

**DOI:** 10.1371/journal.pone.0094770

**Published:** 2014-04-15

**Authors:** Lan Mu, Yinli Liang, Ruilian Han

**Affiliations:** 1 Institute of Soil and Water Conservation, Northwest A&F University, Yangling Shaanxi Province, China; 2 Institute of Soil and Water Conservation, Chinese Academy of Sciences and Ministry of Water Resources, Yangling, Shaanxi Province, China; Agroecological Institute, China

## Abstract

The conversion of farmland to forestland not only changes the ecological environment but also enriches the soil with organic matter and affects the global carbon cycle. This paper reviews the influence of land use changes on the soil organic carbon sink to determine whether the Chinese “Grain-for-Green” (conversion of farmland to forestland) project increased the rate of SOC content during its implementation between 1999 and 2010 in the hilly and gully areas of the Loess Plateau in north-central China. The carbon sink was quantified, and the effects of the main species were assessed. The carbon sink increased from 2.26×10^6^ kg in 1999 to 8.32×10^6^ kg in 2010 with the sustainable growth of the converted areas. The black locust (*Robinia pseudoacacia L.*) and alfalfa (*Medicago sativa L.*) soil increased SOC content in the top soil (0–100 cm) in the initial 7-yr period, while the sequestration occurred later (>7 yr) in the 100–120 cm layer after the “Grain-for-Green” project was implemented. The carbon sink function measured for the afforested land provides evidence that the Grain-for-Green project has successfully excavated the carbon sink potential of the Shaanxi province and served as an important milestone for establishing an effective organic carbon management program.

## Introduction

Soil organic carbon (SOC) is an important component of soil that plays a key role in the functions of both natural and agricultural ecosystems. In ecosystem services, SOC is critical for ensuring sustainable food production owing to its nutrient retention function and water-holding capacity [1], [2]. The global SOC stock has been estimated to be 1400–1500 Pg C in the upper 100 cm soil layer [3]–[5], which is approximately twice the amount of C in the atmosphere and three times the amount stored in terrestrial vegetation [6]. Todd-Brown et al [7] reported that the present-day global SOC stocks range from 514 to 3046 Pg C among 11 earth system models (ESMs). Thus, slight reductions in SOC contents due to changes in land-use, soil management, or rates of soil erosion, could significantly raise the CO_2_ in the atmosphere. Due to natural drought conditions, intensive human disturbance and severe soil erosion, the hilly-gully area of the Loess Plateau has the lowest soil organic carbon density (SOCD) in China [8]. However, it is possible to increase the organic carbon content and carbon sequestration capacity in the soils of this region through appropriate reforestation of degraded sloping croplands and other ecosystems, whose resilience capacity is intact [1], [9].

Changes in land use may alter the land cover patterns in ways that can impact the biomass and soil carbon stocks [10] and alter the rate of C input and output to the soil, ultimately changing the soil C content [11]. Soil C sinks are not permanent and often persist only as long as appropriate management practices (conservation tillage and erosion control) are maintained. When a land-management or land-use change is reversed, the C accumulated as a result of the change is lost, usually more rapidly than it was accumulated [12]. And greater attention to the possibility of encouraging the growth of the forests as a means of removing carbon dioxide (CO_2_) from the atmosphere [13]. Several studies have addressed the effects of the conversion of farmland to forestland on the SOC. In a long-term experiment, Guo and Gifford [14] showed that the conversion of forestland or grassland to farmland resulted in significant SOC reduction; the conversion of forestland to both farmland and grassland did not lead to SOC reduction in all cases, but always resulted in significant carbon sequestration from the atmosphere. Ostle et al [15] also found that soil carbon losses occur when grasslands, managed forest lands or native ecosystems are converted to croplands, and soil carbon gains are made when croplands are converted to grasslands, forest lands or native ecosystems. Carbon stored in forest ecosystems represents a substantial portion of the global C stock; worldwide, forests contain ∼70% of all plant C and ∼20% of all soil C [16]. The conversion of farmland to forestland is therefore an effective method for preventing soil erosion, which also has a large net sink effect on the SOC storage in the hilly-gully areas of China. However, the balance between inputs of organic matter, primarily from vegetation, and losses, as a result of decomposition, leaching and erosion, determines the magnitude of the carbon reservoir of the hilly-gully areas of China. The net CO_2_ released via soil and water loss is calculated to be 8.4 g C m^−2^ yr^−1^ [17].

The hilly-gully areas of the Chinese Loess Plateau are known for their agricultural history and severe soil erosion [18]. Since the 1950s, the Chinese government has made substantially efforts to control the soil erosion and restore vegetation to the region. For example, an extensive tree-planting project was undertaken in the 1970s, and integrated soil erosion control was performed on the watershed scale in the 1980s and 1990s. Despite these efforts, the soil erosion remained unchecked, and vegetation had not been substantially restored to the region by the late 1990s [10]. In 1999, an extensive ecological rehabilitation program known as “Grain-for-Green” (conversion of farmland to forestland) was initiated by the Chinese government; this program is currently the largest land retirement project undertaken in the developing world. The Grain-for-Green program was initially intended to reduce soil erosion and desertification and increase the forest cover in China by retiring steep sloping and marginal lands from agricultural production in the hilly-gully area of the Loess Plateau [19]. The program has now been operating for about 10 years and the natural environment in parts of the Loess Plateau is improving as annual crops are replaced by perennial plants. However, limited attention has been paid to the effect of land use conversion on soil carbon sequestration in the Loess Plateau [20], [21]. The Grain-for-Green program has potential for C gain through the improved land use conversions [[19] and we hypothesized that the conversion of farmland to forestland is one method that may increase SOC storage in soils in this study region in Loess Plateau.

Therefore, the objective of this paper was to assess the changes in the soil organic carbon content and carbon sink benefit following the implementation of the Grain-for-Green project on the Loess Plateau between 1999 and 2010. The paper also discussed the variation of the soil organic carbon content with the type of vegetation and soil age in the study area and assesses the effect of the land cover change project.

## Material and Methods

### Research region

Zichang County was located in the northern Shaanxi Province of the Loess Plateau in China within latitudes 36°59′–37°30′N and longitudes 109°11′–110°01′E. The region had a semiarid continental monsoon climate with a mean annual temperature of 9.1°C and mean annual precipitation of 514.7 mm. Over 70% of the precipitation occurred between June and September. The landform had the typical hilly-gully topology of the Loess Plateau, and the valleys and ravines accounted for approximately 94.6% of the total area, in which the gully density was 8.1 km km^−2^. Soils in the research area originated from parent material of calcareous loess, with a low SOC concentration of less than 10 g kg^−1^. These belong to the Calcic Cambisol group according to the Food and Agriculture Organization and United Nations Education Scientific and Cultural Organization (FAOUNESCO) soil classification system [10]. The soil type was loessal soil with a pH value of 8.6. The soil bulk density was 1.4 g cm^−3^ and total N content was 0.4 g kg^−1^. The area of the croplands was 11.08×10^4^ ha, accounting for 46.24% of the total land area. In this area, agricultural land occupied 39.5% of the area on slopes exceeding 25° (Table 1); this cultivated steep carried soil load of approximately 229.4×10^4^ t yr^−1^ and a water loss of up to 857.8×10^4^ m^3^ yr^−1^. The natural vegetation had been largely destroyed by deforestation and cultivation. Slope land reclamation and cultivation along the slope was the primary cause of soil and water loss.

**Table 1:** The area of the cultivated slope land classification of the ZiChang county.

slope (degree)	area	percentage
	hm^2^	%
<5	17927.87	16.9
5∼10	3050.33	2.9
10∼15	9950.33	9.4
15∼25	33247.4	31.3
25∼35^a^	32532.93	30.7
>35	9362.93	8.8

a The slopes of greater than 25°were cultivated for agriculture occupied 39.5%.

**Figure 1:**
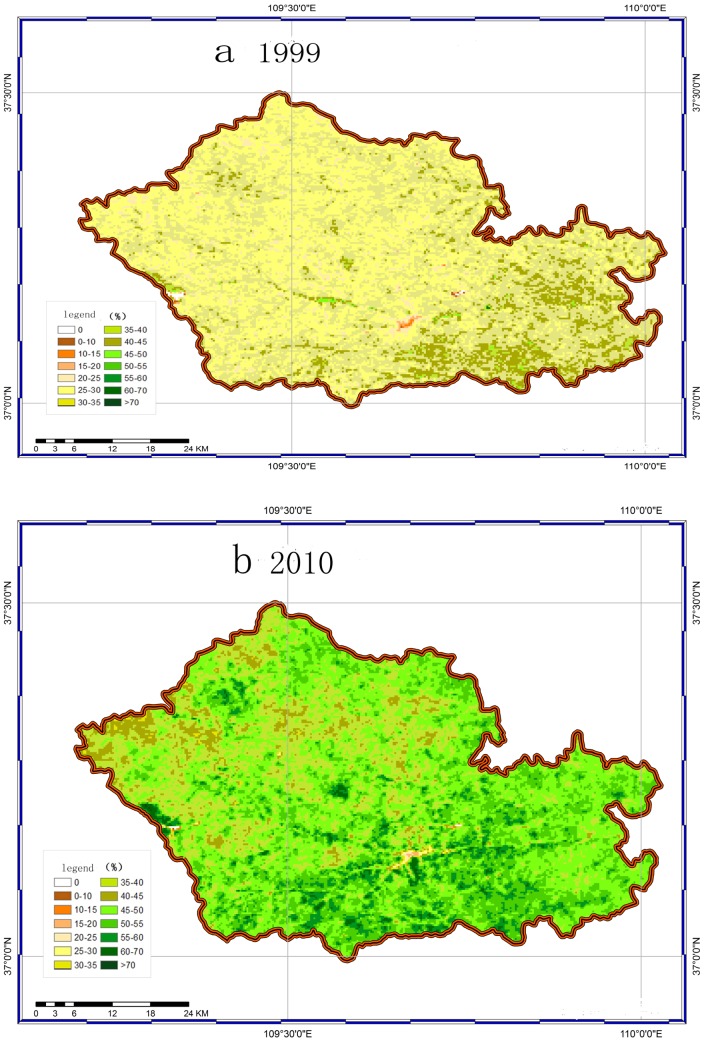
The images of the differences in the remote sensing vegetation coverage between 1999 and 2010 of ZiChang county.

Land degradation, desertification, soil erosion and declining soil fertility had degraded the environment and severely limited the crop yield. In addition, the SOC concentrations in these areas were less than 10 g C kg^−1^. Consequently, an extensive ecological rehabilitation program known as “Grain-for-Green” was initiated in 1999 in the Loess Plateau region. Zichang was the demonstration county for the larger Shaanxi project. The program in the county had now been implemented for approximately 10 yr (since 1999), and the natural environment was improving as annual crops were replaced by perennial plants and as the vegetation cover increases. The vegetation coverage rate had increased from 6.16% to 32.8%, and the greening rate in the county had reached 62% (Figure 1). And the vegetation reduced the soil and water lost with the amount of approximately 27.5×10^4^ t yr^−1^ and 102.9×10^4^ m^3^ yr^−1^, respectively (the data come from the Bureau of Soil and Water conservation, Zichang county). These changes had significantly improved the ecological environment, reduced soil and water loss and produced a carbon sink effect in the area [22].

### Land cover changes between 1998 and 2010

**Figure 2:**
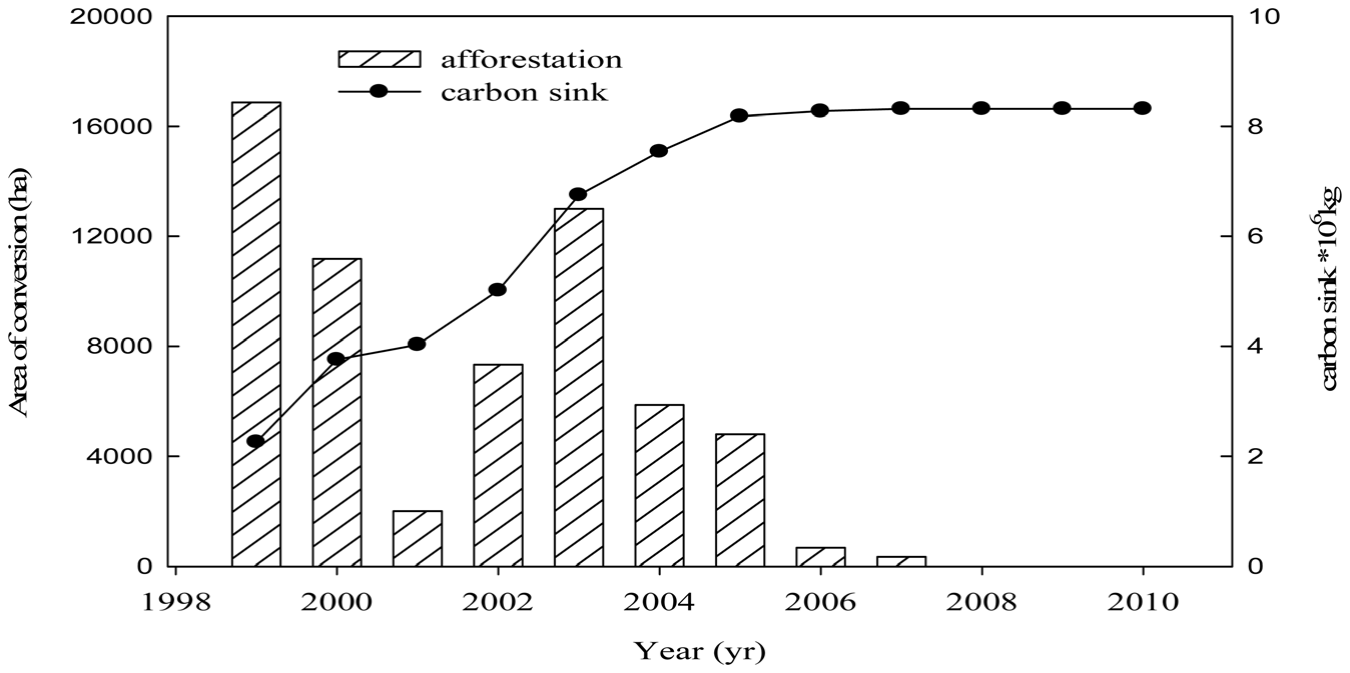
The areas of land cover change (farmland to forestland) and the carbon storage from 1998 to 2010.

The main land use change in the Zichang region was the ongoing conversion of cropland to forestland between 1999 and 2010, with the afforestation area occupying up to 6.2×10**^4^** ha. Figure 2 showed the area over which the land cover was altered during the 10-yr period. 2 sites were selected for the study on the regional scale and a multi-year field study was conducted in the experimental sites at the Zichang experimental station, the institute of soil and water conservation, Chinese Academy of Science. The black locust (*Robinia pseudoacacia L.*) and alfalfa (*Medicago sativa L.*) were the main plantation species during this period.

#### Site A: alfalfa

Alfalfa is a perennial flowering plant in the pea family Fabaceae cultivated as an important forage crop in many countries around the world. Its root nodules contain bacteria, *Sinorhizobium meliloti* with the ability to fix nitrogen, producing a high-protein feed regardless of available nitrogen in the soil. It has been widely cultivated for restoration in Loess Plateau because of its nitrogen-fixing ability and livestock fodder. This site was situated in a hilly - gully slope which faced north, with a slope of 15°. The planting density was 150 plants /m^2^. Three different ages of alfalfa (3, 7 and 12 yr old) were selected for detailed investigation to analyze the effect on the SOC content. The three afforestation chronosequences of alfalfa selected in the same site and the only difference between them is the time when they had been afforested. Each chronosequences of alfalfa had three replications and the total were 3 ages×3 replications = 9 plots.

#### Site B: black locust

Black locust is a tree of the genus Robinia in the subfamily Faboideae of the pea family Fabaceae which is an exotic nitrogen-fixing tree native to Southeastern North America. It has been widely cultivated for restoration because of its drought resistance, high survival rate, ability to improve the soil nutrient status and remarkable growth rate [23]. At the present, it is the most widely cultivated species in the region. The black locust site was situated in a hilly - gully slope which faced north, with a slope of 19°. And the stand density of black locust was 1667 stands/hm^2^. Four afforestation chronosequences of black locust (3, 7, 12 and 15 yr old) were selected for detailed investigation to analyze the effect on the SOC content. The four afforestation chronosequences of black locust selected in the same site and the only difference between them is the time when they had been afforested. Each chronosequences of black locust had three replications and the total were 4 ages×3 replications = 12 plots.

### Calculation of forest carbon sink

Plenty of calculation methods for forest carbon sink have been evolved by experts and scholars such as carbon density method, carbon balance model F-CARBON and so on, which are precise but trivial and fall into the category of traditional nature science. In this research, the carbon sink was calculated on the basis of calculation method as forest storage extension suggested by Xi and Li [24] considering of the practicality and maneuverability [25].

Carbon sink was calculated using the following equation: 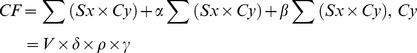



Where S_x_ is the area of forest in the research region; C_y_ is the carbon density of the forest in the research region; V is the volume per unit area of forest; α is the carbon transfer coefficient of undergrowth plants, which is 0.195 in this research; β is the carbon transfer coefficient of forestland, which is 1.244 in this research; δ is the biomass expanding coefficient, which is 0.5 in this research; ρ is volumetric coefficient, which is 1.90 in this research; Υ is carbon content rate, which is 0.5 in this research. Values of each conversion coefficient in calculations of forest carbon sink potential in this region are taken as default values prescribed by intergovernmental Panel on Climate Change [26].

### Soil sample collection and analysis

In November 2010, the soil samples were collected with a 5-cm diameter soil auger and were extracted in 20-cm incremental subsamples, which were subsequently mixed by hand. Soil samples were collected from each soil layer (0–10 cm, 10–20 cm, 20–40 cm, 40–60 cm, 60–80 cm, 80–100 cm and 100–120 cm) at five different locations selected within a 10 m radius surrounding each plot to analyze the soil organic carbon content. The five samples collected for each layer were subsequently mixed by hand, yielding one representative sample for each layer at each site. The total carbon stock for multiple soil layers was calculated by summing the soil carbon stocks of the layers.

All of the soil samples were air-dried, sealed in airtight bags and transported to the Institute for Soil and Water Conservation of the Chinese Academy of Science in Yangling, Shaanxi province to determine the SOC contents. The SOC was determined using the oil bath-K_2_CrO_7_ titration method after digestion, and the soil bulk density (BD) was determined using the ring tube method suggested by the Chinese Editorial Committee for Soil Analysis [27]. Total N was measured by the Kjeldahl procedure and pH was measured by electronic pH-meter [28].

### Statistical analysis

Each SOC contents in composite samples for each plantation species of various depths were averaged at the same ages following afforestation to perform statistical analysis. Analysis of variance was performed using the SPSS 16.0 software. Means were compared by least significant difference (LSD) at p<0.05 or p<0.01 level. A one-way analysis of variance (ANOVA) was used to analyze the effect of each factor (land use, soil depths, ages and species) on the SOC concentration.

## Results

### Carbon sink effects following conversion of farmland to forestland

The forest carbon storage increased continuously as the farmland to forestland conversion project was implemented between 1999 and 2010. The carbon sink increased from 2.3×10^6^ kg in 1999 to 8.3×10^6^ kg in 2010 with the sustainable growth of the converted areas (Figure 2). The total carbon sink increased by 268% compared to that in the first yr of implementation of the project. This result established that the sequestration of carbon (C) is another important environmental effect of afforestation. The land use policy affected the type, distribution, productivity and turnover of vegetation. This policy was therefore a key determinant of whether the land surface was a sink or source of carbon. Forestland had great carbon sequestration potential, especially compared to farmland. The carbon storage induced by the conversion of farmland to forestland in Zichang County was very large.

### SOC content for alfalfa and black locust based on the afforestation chronosequence

**Figure 3:**
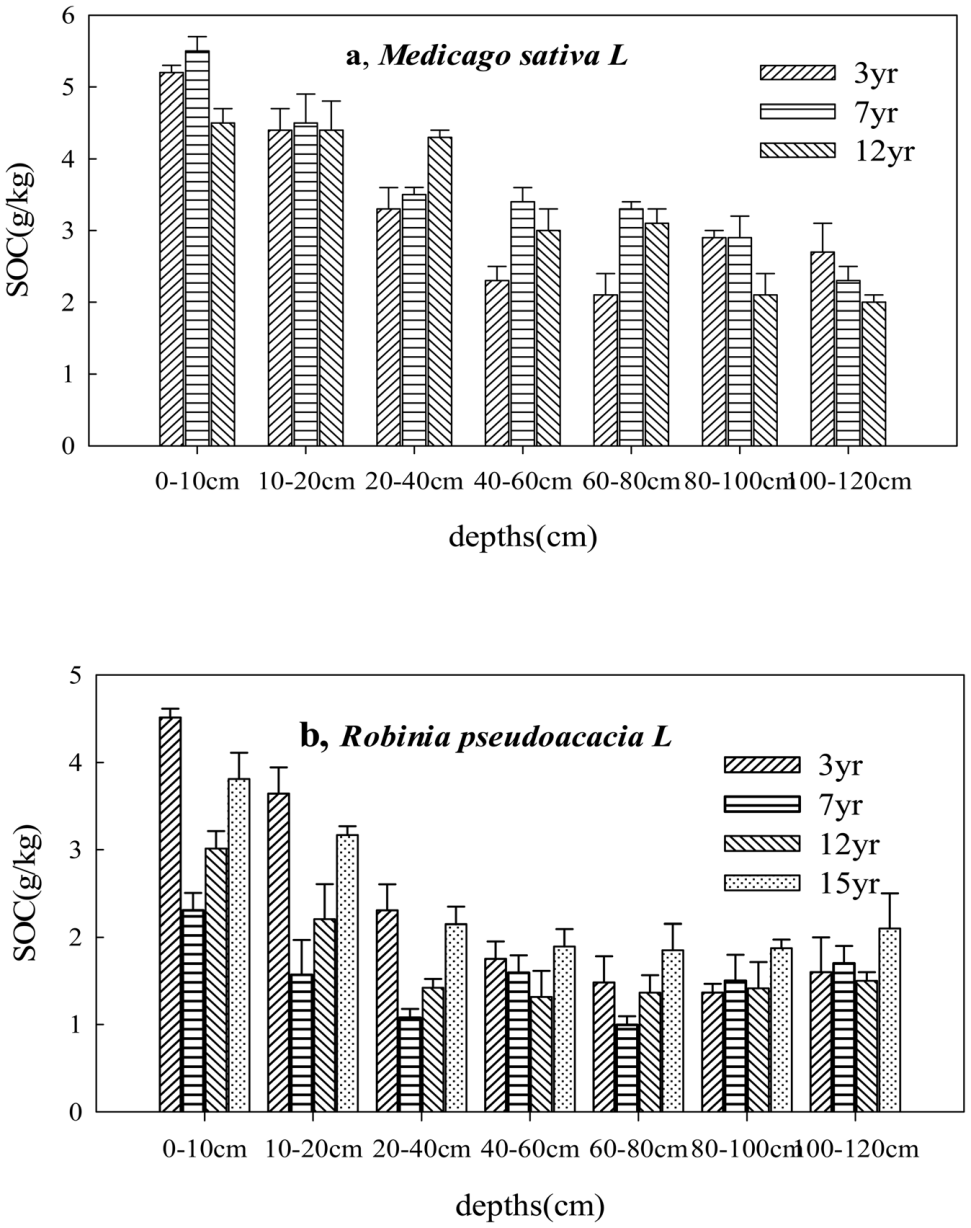
Changes of SOC sequestrations at different ages under alfalfa (*Medicago sativa L*) and black locust (*Robinia pseudoacacia L*) for each depth.

In the alfalfa soil, there was a significant increase in the SOC content in the 0–20 cm and 40–100 cm layers with increasing age (p<0.01; Figure 3a) from 3 yr to 7 yr. Subsequently, the SOC content decreased slightly with increasing age (from 5 to 4.5 g kg^−1^ and from 3.2 to 2.4 g kg^−1^ in the last 5 yr). The SOC did not change appreciably in the 20–40 cm layer (Figure 3a). In contrast with the soil samples from <100 cm depth, the changes in the soil C in the 100–120 cm layer were significantly correlated with plantation age; the SOC decreased continuously with age from 2.7 to 2.0 g kg^−1^.

The SOC exhibited a different trend with age in the black locust soil compared to the alfalfa soil (Figure 3b). The SOC content decreased in the first 7 yr at depths below 100 cm, from 3.0 to 1.7 g kg^−1^ (for yr 4–7, the concentration was consistently lower than that measured for the first 3 yr). Interestingly, the C content then exhibits a gradually increasing trend between 7 and 15 yr (Figure 3b) reaching 3.8 g kg^−1^ at an age of 15 yr. In contrast, the ecosystem C stores in the 100–120 cm layer of the old plantation were clearly higher than those in the younger afforestation sites, which had increased by 31.3% 15 yr after afforestation. The SOC content in the two afforestation plantations therefore provide evidence for a carbon sink once the farmland was converted to forestland.

### A comparison of the SOC contents for plantation species of various depths at the same ages following afforestation

The statistical comparisons in the ANOVA demonstrate that the dependence of the SOC on depth in the areas converted to forestland is significant at a level of p<0.05 (Table 2). Between ages of 7 and 12 yr, the SOC content in the alfalfa soil decreases with increasing depth, and the SOC content in the 0–60 cm layer exceeds that at depths above 60 cm. The soil C content is greatest (5.5 and 4.5 g kg^−^1) in the 0–10 cm layer. However, in the first 3 yr, the SOC decreases significantly (p<0.01, Table 2) with increasing depths of 0–80 cm and then exhibits a smaller increase at depths of 80–100 cm. For black locust, the SOC decreases continuously at depths of 0–100 cm and then increases slightly in the 100–120 cm layer in the 3-, 7-, 12- and 15-yr-old forests. Although the C concentration trend based on depth was different for the two plantations, the SOC content in the topsoil was significantly greater (p<0.01, Table 2) than that in the subsoil for both plantations.

**Table 2:** SOC^a^ at the different depth of the alfalfa (*Medicago sativa L*) and black locust (*Robinia pseudoacacia L*) following the afforestation.

Depth (cm)	alfalfa (*Medicago sativa L*) (g kg^−1^)				black locust (*Robinia pseudoacacia L*) (g kg^−1^)				
	3 yr	7 yr	12 yr	mean	3 yr	7 yr	12 yr	15 yr	mean
0–10 cm	5.2a	5.5a	4.5a	5.1a	4.5a	2.3a	3.0a	3.8a	3.0a
10–20 cm	4.4b	4.5b	4.4a	4.5a	3.6b	1.6b	2.2ab	3.2b	2.3b
20–40 cm	3.3c	3.5c	4.3a	3.8b	2.3c	1.1c	1.4bc	2.1c	1.5c
40–60 cm	2.3ef	3.4cd	3.0b	2.9c	1.8d	1.6b	1.3c	1.9c	1.6c
60–80 cm	2.01f	3.3cd	3.1b	2.8c	1.5d	1.0c	1.4bc	1.9c	1.4c
80–100 cm	2.9cd	2.9d	2.1c	2.6c	1.4d	1.5b	1.4bc	1.9c	1.6c
100–120 cm	2.7de	2.3e	2.0c	2.3c	1.6d	1.7b	1.5bc	2.1c	1.8cb

a Data in the column are mean values (n = 3 for alfalfa and black locust), which are compared among different depths within each ages and are not different at the

5% level of significance if followed by the same letter.

**Figure 4:**
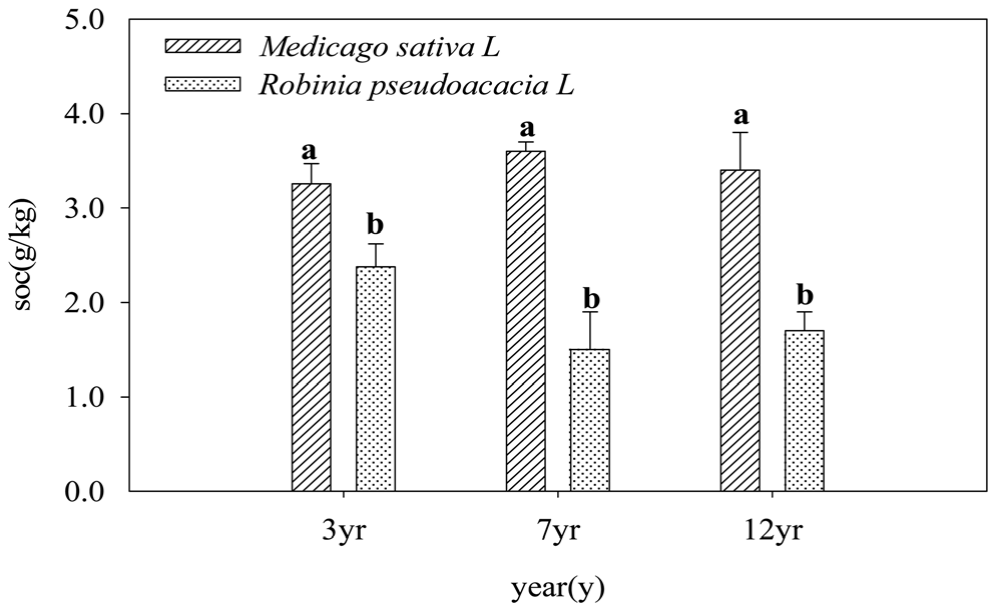
The difference of SOC between alfalfa (*Medicago sativa L*) and black locust (*Robinia pseudoacacia L*) at the same depths under the different ages at the 0–120 cm depths under the 3,7 and 12 ages after the conversion of farmland to forestland.

To inform organic carbon management strategies, the dependence of the SOC content on the type of vegetation is also quantified (Figure 4). The SOC concentration in the alfalfa soil exceeded that for the black locust soil at a given age in the 0–100 cm and 100–120 cm layers. The soil C for the two plantations shows a highly significant difference between alfalfa and black locust (p<0.05 or p<0.01, Figure 4). The SOC in the 3-yr-old alfalfa was approximately 36.9% higher than that in the black locust in the 0–100 cm layer. The largest difference between the two species was measured for the 7-yr-old plantation; the SOC contents for this plantation exhibit highly significant differences between the two species at each depth (p<0.01, Figure 4). The soil C is similar for both species on the 12-yr-old plantation and exhibits a significant difference (p<0.05, Figure 4) compared to the 7-yr-old plantation at each depth.

### Relationship between the SOC content, depth and other factors as a function of age following afforestation

**Figure 5:**
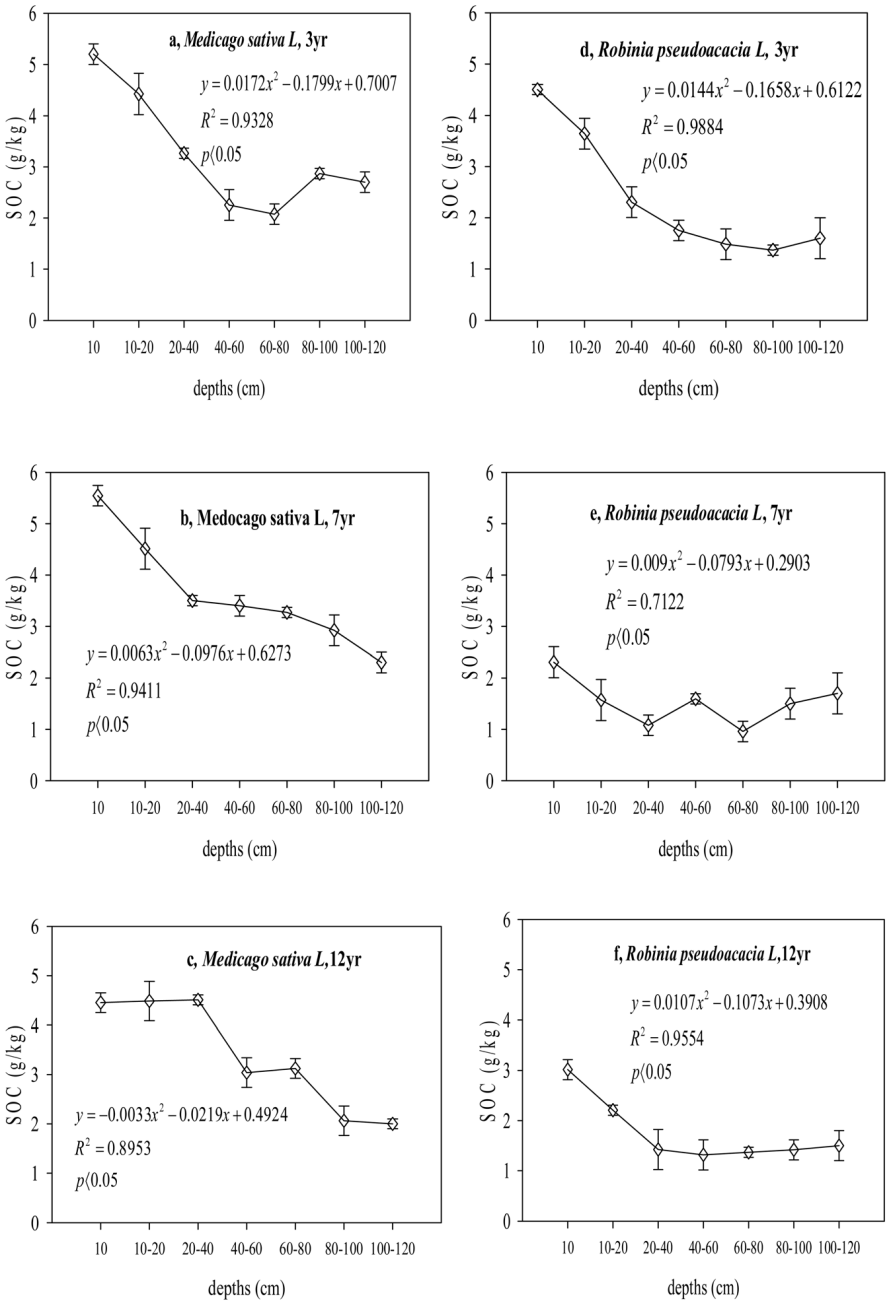
Relationship between the SOC and depths of the alfalfa (*Medicago sativa L*) and the black locust (*Robinia pseduoacacia L*) under 3, 7 and 12 years old following afforestation.

A change in land use from farmland to forestland and grassland implies that the annual cycle of cultivating and harvesting crops is replaced by the much longer forest cycle. Therefore, many factors influence the SOC in the alfalfa and black locust soils after the land cover changes. The relationships between the SOC content, soil depth and age are summarized in Figure 5. For the 3-yr-old afforested land, there is a strong correlation (R^2^ = 0.93, R^2^ = 0.99, p<0.01; Figure 5a, b) between SOC content and depth. As shown in Fig 5, the same trend is obtained for 7- (Figure 5c, d) and 12-yr-old (Figure 5e, f) soil. The SOC is related to the afforestation yr, with the SOC decreasing gradually with increasing age (Figure 5).

## Discussion

### Effect of the farmland-to-forestland conversion project on the SOC stocks

Land use change can cause a change in land cover and an associated change in the carbon stocks [29]. Each soil has a given carbon carrying capacity, i.e., an equilibrium carbon content depending on the nature of the vegetation, precipitation and temperature [30]. The equilibrium carbon stock arises from a balance between the inflow and outflow to the carbon pool [31]. The equilibrium between the carbon inflow and outflow to the soil is disturbed by any land change until a new equilibrium is reached in the new ecosystem. In our study, we have found that in the farmland-to-forestland conversion project, the soil acts as a carbon sink (from 2.26×10^6^ kg in 1999 to 8.32×10^6^ kg in 2010). Other studied has got the same results. It has also been estimated that UK forestry and grassland sequester 110±4 kg and 240±200 kg of carbon per hectare yr, respectively, whereas croplands lose on average 140±100 kg of carbon per hectare per yr [32]. Ostle et al [15] indicated that soil carbon accumulates more slowly (decadal) but gains can be made when croplands are converted to grasslands, plantation forest or native woodland. The results of this study partially support the hypothesis that the afforestation of formerly arable land leads to increased C storage in the soil over the short term (approximately 30 yr).

The balance between the inputs (primarily from vegetation) and losses of organic matter (as a result of decomposition, leaching and erosion) determines the magnitude of the area's land carbon reservoir. The net effect of the afforestation of cultivated slope land on the soil C content depends not only on the new C gained, but also on the C lost as a result of the previous management. Although the vegetation carbon stock in the hilly-gully areas are relatively small, plant matter is the single most important source of carbon input to the soil [33]. Therefore, the higher SOC content in the two plantations in our study could be a result of primarily the greater annual litter input and fine root biomass compared to the cropland.

### SOC accumulation for different depths and ages following the afforestation of the cropland

The soil organic carbon content increase in the topsoil (0–40 cm) for both plantations (from 3.8 to 5.1 g kg^−1^ and 1.5 to 3.0 g kg−1) after the conversion of cropland into the forest, is in consistent with other findings for the Loess Plateau [34], [10] and other areas of the world [14], [35]–[37] suggesting that the SOC may be highly sensitive to variations in the soil moisture, soil temperature and/or litter input (all of which depend strongly on depth). The increase in the topsoil SOC may be associated with the higher carbon input and lower rates of SOC decomposition and soil erosion associated with alfalfa plantations and black locust forest. It is well established that the top soil is the part of the soil profile that is most susceptible to land use change and disturbance, such as plowing and drainage. The increase in SOC decomposition resulting from tillage disturbance is a key reason for the decrease in SOC following the cultivation of perennial ecosystems [38]. Within limits, soil C increase with increasing soil water and decreasing temperature and the top soil of the increasing water within temperature zones can increase plant production and increase plant litter and root production [39]. In contrast, the cessation of tillage during the establishment of forest on cropland can reduce the SOC decomposition rate, thus increasing the SOC content. Furthermore, the addition of nitrogen derived from nitrogen-fixing species (in this case, alfalfa and black locust) has been well documented and can lower the decomposition of old and new carbon [40], hence favoring SOC sequestration. Moreover, the forest is widely found to be less susceptible to soil erosion compared to the cropland on the Loess Plateau [41], which may contribute to the higher topsoil SOC content following the afforestation of the cropland. And there is also an increase in the carbon input from aboveground litter and fine root biomass in the alfalfa and black locust, which may lead to SOC accumulation during the afforestation.

Understanding the dependence of the SOC content on the plantation age is important for the development of improved biological soil management practices on both local and national scales. This understanding will also help to identify different species that are ecologically compatible and economically useful. The SOC stores in the younger plantation were clearly higher than those in the old afforestation sites, which exhibited decreases of 15.6% and 50% for alfalfa and black locust 12 yr after afforestation. However, for black locust, the SOC continues to increase gradually 15 yr after the conversion of farmland to forestland. A fundamental assumption regarding the effect of land change on the carbon sink is that younger, growing forests sequester carbon more rapidly than older forests. For several yr following a disturbance, the land becomes a carbon source, but as it matures, it becomes a large carbon sink, which slowly declines with age during late succession [42], [43]. This trend may owe to the past application of organic fertilizer when the land was used as cropland, producing a higher initial soil C content just before afforestation. When agricultural land is no longer used for cultivation and is allowed to revert to natural vegetation, the balance of C input and output in the agricultural system is broken, as no fertilizer is added to the afforested land.

### Factors affecting the SOC sequestration in the “Grain-for-Green” project

The contribution of afforestation to the SOC improvement is controlled by several factors, including the severity of the land degradation, plant species and composition, climate, duration, land use history and management. The effect of the Grain for Green project on SOC sequestration was mainly influenced by ages and depths, which explained 43.2% and 35.6% (Table 3) of the variation of SOC. vegetation species explained 21.2% (Table 3) of the variation in SOC. Xu et al [44] found that the effect of the “Grain-for-Green” project on the SOC sequestration was influenced primarily by the land use type and age, which explained 55.6% and 24.1% of the variation in the SOC, respectively. The SOC content therefore varies across plantations because the C sequestration is affected by the varying land management. Our findings on the carbon sink induced by the project and the relationship between the SOC content and the ages following afforestation supported the conclusion of Xu et al [44]. Topographic factors such as the slope aspect and position were also found to explain 8.5% of the variation in the SOC [44]. Reay ea al [45] indicated that by increasing the productivity, nitrogen enrichment of some natural ecosystems may enhance their capacity to sequester carbon. Clearly, we demonstrate that afforestation has a positive influence on the SOC concentration in our study, depending on the species planted (alfalfa has a higher SOC content than black locust). The species-induced difference in the SOC content and other soil properties may owe to a difference in biomass production and nutrient cycling via litter fall and root turnover [46].

**Table 3:** Contribution of different factors to the variation of SOC content (variance components, n = 45).

	Variance Source		
SOC content	Afforestation ages	Depths	Plantation Species
Variance	16.62	13.69	8.15
Variance percentage to total variance (%)	43.20	35.60	21.20

## Conclusion

We examined the changes in the carbon sink and SOC content of the topsoil and subsoil following the “Grain-for-Green” (farmland-to-forestland conversion) project. The afforested land (alfalfa and black locust) soil displayed remarkable SOC sequestration compared to the sloping croplands. The two plantations studied exhibited SOC sequestration potential in the top soil (0–100 cm) in the initial 7-yr period, while the sequestration occurred later in the 100–120 cm layer (>7 yr after the “Grain-for-Green” project was implemented). Furthermore, our analyses provide evidence that SOC can be influenced by afforestation in semi-arid region such as the Loess Plateau, China. From a regional perspective, type of land use, age and species all had significant effects on the SOC sequestration in the “Grain-for-Green” project. The implementation of the program has further enhanced the vegetation restoration and ecological conservation and strengthened the management of the Loess Plateau ecosystem.

## Supporting Information

Figure S1 Editorial certificate.
(PDF)
